# Safety and efficacy of uninterrupted treatment with edoxaban or warfarin during the peri‐procedural period of catheter ablation for atrial fibrillation

**DOI:** 10.1002/joa3.12351

**Published:** 2020-04-26

**Authors:** Kazuya Naito, Masataka Nakano, Atsushi Iwasa, Yoshio Maeno, Yoshiaki Shintani, Takeshi Yamakawa, Kotaro Miyashita, Keishiro Oyama, Daisuke Nakai, Masaya Katagiri, Hideaki Kido, Shinichiro Masuda, Keiichi Kohashi, Tetsuya Kawamata, Shuzou Tanimoto, Naoki Masuda, Nobuhiko Ogata, Takaaki Isshiki

**Affiliations:** ^1^ Department of Cardiology Ageo Central General Hospital Saitama Japan; ^2^ Department of Cardiology New Tokyo Hospital Chiba Japan

**Keywords:** atrial fibrillation, catheter ablation, edoxaban, uninterrupted anticoagulation, warfarin

## Abstract

**Background:**

The real‐world safety and efficacy of uninterrupted anticoagulation treatment with edoxaban (EDX) or warfarin (WFR) during the peri‐procedural period of catheter ablation (CA) for atrial fibrillation (AF) are yet to be investigated.

**Methods:**

We conducted a two‐center experience, observational study to retrospectively investigate consecutive patients who underwent CA for AF and received EDX or WFR. We examined the incidence of thromboembolic and bleeding complications during the peri‐procedural period.

**Results:**

The EDX and WFR groups included 153 and 103 patients, respectively (total: 256 patients). Demise or thromboembolic events did not occur in either of the groups. The incidence of major bleeding in the EDX and WFR groups was 0.7% and 2.9%, respectively. The total incidence of major/minor bleeding in the EDX and WFR groups was 7.8% and 8.7%, respectively. Of note, the incidence of bleeding complications in the uninterrupted WFR strategy group was markedly high in patients with an estimated glomerular filtration rate (eGFR) <30 (75%) or a HAS‐BLED score ≥3 (60%). Patients with eGFR ≥30 and a HAS‐BLED score ≤2 had a lower incidence of bleeding (<10%), regardless of the administered anticoagulation drug (EDX or WFR).

**Conclusions:**

This study confirmed the safety and efficacy of uninterrupted anticoagulation therapy using EDX or WFR in real‐world patients undergoing CA for AF. Patients with severely impaired renal function and/or a higher bleeding risk during uninterrupted therapy with WFR were at a prominent risk of bleeding. Therefore, particular attention should be paid in the treatment of these patients.

## Graphical abstract

Patients with severely impaired renal function and/or a higher bleeding risk during uninterrupted therapy with Warfarin were at a prominent risk of bleeding complications. Therefore, utmost attention should be paid in the treatment of these patients to prevent bleeding complications.

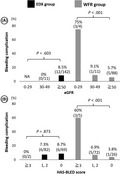

**Abbreviations**
ACT: activated clotting time
AF: atrial fibrillation
CA: catheter ablation
DOAC: direct oral anticoagulant
eGFR: estimated glomerular filtration rate
PT‐INR: prothrombin time‐international normalized ratio

## 1 INTRODUCTION

Atrial fibrillation (AF) is one of the clinically relevant arrhythmias commonly encountered in daily clinical practice.^1^ Owing to an increased risk of thromboembolism, anticoagulation treatment is recommended for patients with AF in the European and Asia Pacific countries, including Japan.^2^ Vitamin K antagonists, such as warfarin (WFR), have been used for a long time to prevent thromboembolic events in patients with AF. However, the benefits of this treatment are occasionally counterbalanced by the risk of bleeding.^3^ Recently, the use of direct oral anticoagulants (DOACs) is rapidly spreading as an alternative to WFR. DOACs exert anticoagulant effects regardless of a vitamin K intake. Randomized, controlled clinical trials for the prevention of stroke in patients with AF demonstrated that DOACs are at least as safe and effective as vitamin K antagonists. One such study, the ENGAGE AF‐TIMI 48 (Effective Anticoagulation with Factor Xa Next Generation in AF‐Thrombolysis in Myocardial Infarction 48) trial, demonstrated the noninferiority of edoxaban (EDX) to WFR based on the low incidence of stroke, systemic embolism, and bleeding.^4^

Apart from pharmacological therapy, catheter ablation (CA) is another therapeutic option for AF.^5^ However, the risk of fatal bleeding and thrombosis during the peri‐procedural period of CA often results in controversy regarding the optimal usage of anticoagulation drugs.^6^ Thus far, only a few studies have assessed the safety and efficacy of EDX during the peri‐procedural period of CA worldwide.^7^ Therefore, we sought to investigate the incidence of bleeding and thromboembolic complications of uninterrupted treatment with EDX during the peri‐procedural period of CA, vs those of WFR in the real‐world Japanese population.

## 2 METHODS

### 2.1 Study subjects

This study complied with the tenets of the Declaration of Helsinki, and a locally appointed ethics committee approved the research protocol. We conducted a two‐center experience, observational study to retrospectively investigate consecutive patients who underwent CA for AF and received anticoagulation treatment with WFR or EDX at the New Tokyo Hospital and Ageo Central General Hospital between January 2015 and December 2018. The decision to proceed with CA was based on the consensus of arrhythmia specialists according to the guidelines of the Japanese Circulation Society.^8^ Clinical information regarding the patient characteristics and conditions was acquired from the medical records. The CHADS_2_ and HAS‐BLED scores were calculated as previously described.^9^, ^10^ Peri‐procedural complications, such as major/minor bleeding and thromboembolic episodes, were also evaluated from the medical records. Major bleeding was defined based on the criteria established by the International Society on Thrombosis and Haemostasis.^11^ Minor bleeding was defined as nonmajor bleeding that did not necessitate a surgical procedure or blood transfusion, but necessitated intervention such as a manual compression, additional resting time, laboratory testing or image inspection. The observational period for peri‐procedural complications was 1 month prior to and 2 months after the procedure. The selection of anticoagulation treatment was at the physicians' discretion. Patients who received treatment with EDX were classified in the EDX group. For those with a creatinine clearance level <50 mL/min, low body weight (<60 kg) or concomitant use of potent P‐glycoprotein inhibitors, a low dose (30 mg) of EDX was administered (39.2%). The remaining patients (60.8%) received a high dose (60 mg) of EDX. On the day of the CA procedure, EDX was administered within 8 hours prior to or after the procedure, based on the operators' discretion. Patients who received treatment with WFR were classified in the WFR group. For patients aged >70 years in this group, WFR was adopted to maintain a prothrombin time‐international normalized ratio (PT‐INR) of 2.0‐3.0 or 1.6‐2.6.^8^ The administration of WFR was continued on the procedural day, except when the PT‐INR exceeded the target range. In both groups, anticoagulation therapy was administered for at least 4 weeks prior to and 2 months after the procedural day and was continued on the procedural day.

### 2.2 CA procedure

Transesophageal echocardiography was performed in most patients (97%) on the day prior to the procedure. Dexmedetomidine hydrochloride, pentazocine, and fentanyl citrate were used as intravenous anesthetics. A bolus of unfractionated heparin (80‐120 U/kg) was injected prior to performing the septal puncture. Subsequently, the administration of unfractionated heparin (500‐3000 U) was continued to achieve an active clotting time (ACT) of 300‐350 seconds every 15 minutes during the procedure. CARTO (Biosense Webster Inc) or ENSITE (Abbott) were used as the three‐dimensional mapping system. All patients who underwent initial ablation, and those who underwent secondary ablation because of pulmonary vein reconduction, were subjected to pulmonary vein isolation. Superior vena cava isolation, cavo‐tricuspid isthmus ablation, and left atrial posterior wall isolation were performed in patients in whom nonpulmonary vein foci were induced or additional ablation was deemed necessary by the operator. Following termination of the procedure, hemostatic compression was performed for at least 10 minutes until adequate hemostasis was achieved.

The strategy for radiofrequency CA at the New Tokyo Hospital and Ageo Central General Hospital was as follows: (a) a BeeAT cardioversion catheter (Japan Lifeline) was placed into the coronary sinus through a 7 Fr sheath using the jugular vein approach; and (b) two 8.5 Fr sheaths and one 7 Fr sheath or three 8.5 Fr sheaths were inserted into the femoral vein. A 20‐pole mapping catheter (Lasso, Biosense Webster Inc; PENTARAY, Biosense Webster Inc; or Inquiry Optima, Abbott) and 3.5 mm open irrigated tip catheter (Biosense Webster Inc or Abbott) were used.

The strategy for cryoballoon ablation (Arctic Front Advance™ 28 mm cryoballoon; Medtronic, Inc) was as follows: (a) a BeeAT cardioversion catheter (Japan Lifeline) was placed through a 7 Fr sheath into the coronary sinus using the jugular vein approach; (b) two 8.5 Fr sheaths or one 8.5 Fr sheath and one 7 Fr sheath were inserted using the femoral vein. One sheath was exchanged for a steerable 15 Fr sheath (FlexCath™ Advance; Medtronic, Inc) after performing the septal puncture; and (c) a 4‐pole or 10‐pole catheter was placed for ventricular pacing through the femoral vein.

### 2.3 Statistical analysis

Continuous variables are expressed as the mean ± standard deviation or median (interquartile range). The baseline clinical data were compared between the groups using the Student's *t* test or Mann‐Whitney *U* test. Categorical variables are presented as the frequency and percentage using Fisher's exact test. The odds ratio for the risk of complications was calculated via logistic regression analysis. A value of *P* < .05 denoted statistical significance. All statistical analyses were performed using the SPSS software (IBM SPSS Statistics for Windows, version 25.0; IBM Corporation).

## 3 RESULTS

### 3.1 Patient characteristics

A total of 256 patients who underwent CA for AF between January 2015 and December 2018 were selected. Patients with mechanical heart valves (n = 2), ischemic heart disease receiving dual antiplatelet therapy (n = 1), and kidney failure undergoing hemodialysis (n = 1) were included in the present study. The EDX and WFR groups included 153 and 103 patients, respectively. Overall, the time in therapeutic range for WFR was 73.7% from 2 months before to 3 months after the procedure. In the EDX group, 47 patients (31%) received treatment within 8 hours prior to CA, while the remaining 106 (69%) were treated within 8 hours after CA. The baseline patient characteristics are shown in Table 1. There were no significant differences between the EDX and WFR groups in terms of age, sex, height, and body weight. The rate of a CHADS_2_ score ≥2 was significantly lower in the EDX group than the WFR group (19% vs 31%, respectively, *P* = .026). The patients in the WFR group tended to have a history of congestive heart failure (11% vs 3%, respectively, *P* = .008) and higher levels of B‐type natriuretic peptide (66 [28‐113] vs 37 [16‐73], respectively, *P* = .001) compared with those of the EDX group. The incidence of paroxysmal AF was significantly higher in the EDX group than in the WFR group. Although a higher dose of heparin was required in the EDX group than in the WFR group (13 000 [11 000‐15 000] vs 8000 [7000‐10 000] units, respectively; *P* < .001) to maintain an ACT of 300‐350 seconds, the ACT was significantly shorter in the former group (327 ± 26 vs 336 ± 28, *P* = .012).

**TABLE 1:** Demographics of patients who received uninterrupted anticoagulation treatment with edoxaban or warfarin

	EDX group N = 153	WRF group N = 103	*P* value
Age, years, median (IQR)	66 (55‐72)	68 (60‐73)	.160
≧70	55 (36%)	43 (42%)	.349
<70	98 (64%)	60 (58%)	
Female sex	48 (31%)	26 (25%)	.289
Body weight (kg)	66 ± 13	66 ± 12	.819
≧60	99 (65%)	72 (70%)	.384
<60	54 (35%)	31 (30%)	
Height, cm, (IQR)	165 (159‐172)	166 (158‐171)	.889
Paroxysmal AF	134 (88%)	65 (63%)	<.001
Nonparoxysmal AF	19 (12%)	38 (37%)	
Persistent AF	16 (10%)	21 (20%)	
Long persistent AF	3 (2%)	17 (17%)	
Past medical history			
Hypertension	69 (45%)	48 (47%)	.813
Diabetes mellitus	20 (13%)	25 (24%)	.021
Congestive heart failure	5 (3%)	12 (11%)	.008
Previous stroke/TIA	3 (2%)	7 (7%)	.053
CKD (eGFR <50)	11 (7%)	15 (15%)	.061
CHADS₂ score			
0‐1	124 (81%)	71 (69%)	.026
≧2	29 (19%)	32 (31%)	
HAS‐BLED score			
0‐2	151 (99%)	98 (95%)	.095
≧3	2 (1%)	5 (5%)	
Echographic parameters			
LA diameter, mm, (IQR)	38 (32‐41)	40 (37‐45)	<.001
LVEF, %, (IQR)	62 (58‐66)	62 (54‐65)	.107
BNP, pg/mL, (IQR)	37 (16‐73)	66 (28‐113)	.001
eGFR, mL/min/1.73 m^2^, (IQR)	69 (61‐79)	65 (52‐75)	.014
PT‐INR	N.A	2.2 ± 0.5	N.A
Procedure values			
Mean ACT (sec)	327 ± 26	336 ± 28	.012
Injected heparin dose, U, (IQR)	13 000 (11 000‐15 000)	8000 (7000‐10 000)	<.001
Protamine sulfate use (%)	47 (31%)	27 (27%)	.464

Abbreviations: ACT, activated clotting time; AF, atrial fibrillation; BNP, brain natriuretic peptide; EDX, edoxaban; eGFR, estimate glomerular rate; IQR, interquartile range; LA, left atrium; LVEF, left ventricular ejection fraction; PT‐INR, prothrombin time‐international normalized ratio; TIA, transient ischemic attack.

### 3.2 Complications

The complications which occurred during the peri‐procedural period are shown in Table 2. There was no occurrence of thromboembolic events in either of the groups. The incidence of major bleeding was numerically lower in the EDX group vs the WFR group (0.7% vs 2.9%, respectively); however, the difference did not reach statistical significance (*P* = .306). Major bleeding events observed in the WFR group were exclusively cardiovascular complications: that is, cardiac tamponade in two patients (2%) and false femoral aneurysm in one patient (1%). The only one episode of major bleeding reported in the EDX group, that necessitated a blood transfusion of four units, was nose bleeding caused by the use of an esophageal temperature probe. The incidence of minor bleeding did not significantly differ between the EDX and WFR groups (7.2% vs 5.8%, respectively, *P* = .667). Furthermore, we calculated the odds ratios for the incidence of complications stratified according to the incidence of paroxysmal or nonparoxysmal AF, past history of hypertension, diabetes, congestive heart failure, chronic kidney disease, cerebral infarction, and CHADS_2_ and HAS‐BLED scores. Despite the trend toward superiority of the treatment with EDX noted in patients with comorbidities (hypertension, diabetes), there were no significant differences or interactions found in any of the subgroups (Figure S1).

**TABLE 2:** Incidence of thromboembolic and bleeding complications

	EDX group N = 153	WFR group N = 103	*P* value
Any complication	12 (7.8%)	9 (8.7%)	.798
Thromboembolic event	0 (0%)	0 (0%)	>.99
Major bleeding	1 (0.7%)	3 (2.9%)	.306
Cardiac tamponade	0	2	
False femoral aneurysm	0	1	
Bleeding requiring transfusion	1	0	
Minor bleeding	11 (7.2%)	6 (5.8%)	.667

Abbreviations: EDX, edoxaban; WFR, warfarin.

Table 3 includes the results of a subanalysis conducted to assess whether the timing of EDX administration on the day of CA (pre‐CA or post‐CA) affected the incidence rate of complications. The incidence of major and minor bleeding did not significantly differ between pre‐CA and post‐CA (major bleeding: 2.1% vs 0.0%, respectively, *P* = .307; minor bleeding: 6.4% vs 7.5%, respectively, *P* = .548). However, it should be noted that the only one major bleeding episode recorded occurred in a patient “pretreated” with EDX.

**TABLE 3:** Incidence of complications according to the timing of edoxaban intake

	Pre the CA^a^ N = 47	Post the CA^b^ N = 106	*P* value
Any complication	4 (8.5%)	8 (7.5%)	.533
Thromboembolic event	0 (0%)	0 (0%)	>.99
Major bleeding	1 (2.1%)	0 (0%)	.307
Cardiac tamponade	0	0	
False femoral aneurysm	0	0	
Bleeding requiring transfusion	1	0	
Minor bleeding	3 (6.4%)	8 (7.5%)	.548

Abbreviation: CA, catheter ablation.

^a^ This group consists of the patients who took edoxaban the morning before the catheter ablation.

^b^ This group consists of the patients who took edoxaban within 8 h after the catheter ablation.

**FIGURE 1:**
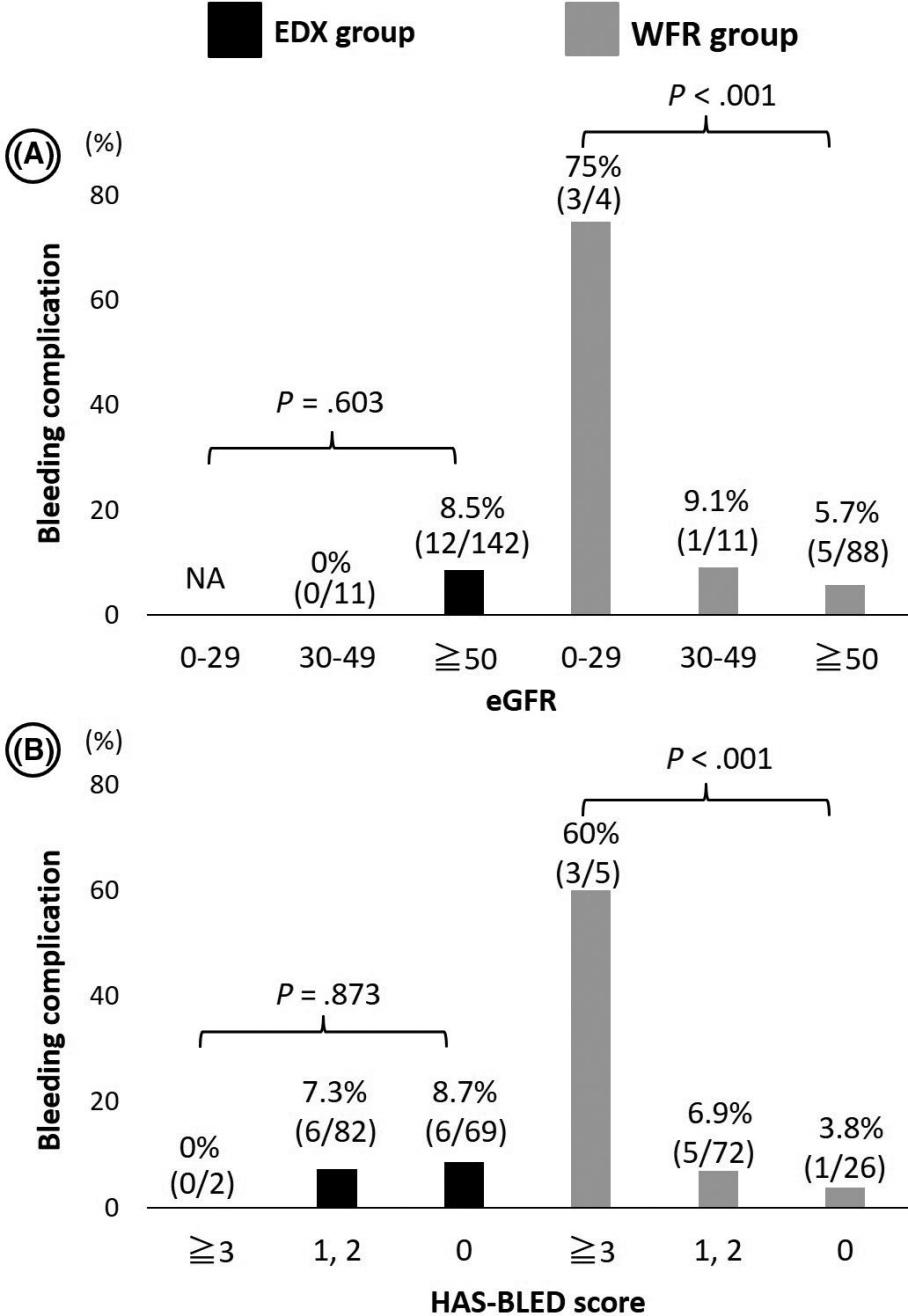
Incidence of major/minor bleeding complications stratified according to the eGFR and HAS‐BLED score. A, The bar graphs represent the incidence of bleeding complications (left, edoxaban group; right, warfarin group) stratified according to the eGFR (≤29, 30‐49, ≥50) in each group. Of note, patients with an eGFR <30 receiving uninterrupted therapy with warfarin were at a prominent risk of bleeding complications. B, The bar graphs represent the incidence of bleeding complications (left, edoxaban group; right, warfarin group) stratified according to the HAS‐BLED score (0, 1‐2, ≥3) in each group. Of note, patients with an HAS‐BLED score ≥ 3 receiving uninterrupted therapy with warfarin were at a prominent risk of bleeding complications. EDX, edoxaban; eGFR, estimated glomerular filtration rate; WFR, warfarin

Subsequently, we sought to investigate the risk factors associated with bleeding complications in the treatment groups (Table 4). In the WFR group, patients with bleeding events had a lower estimated glomerular filtration rate (eGFR) (45 ± 28 vs 67 ± 17, respectively, *P* = .001) and a higher HAS‐BLED score (*P* = .004) compared with those without complications. In contrast, in the EDX group, there were no statistical differences in any parameters. Figure 1 illustrates that the incidence of bleeding complications in the WFR group was markedly high in patients with an eGFR <30 (75%) or a HAS‐BLED score >3 (60%), while those with an eGFR ≥30 and a HAS‐BLED score ≤2 had a lower incidence of bleeding (<10%) regardless of the administered anticoagulation drug. The odds ratio for the risk of bleeding complications in the WFR group patients with an eGFR <30 and/or a HAS‐BLED score ≥3 vs those without was 46.5 (confidence interval: 4.1‐517.2, *P* = .002).

**TABLE 4:** Clinical characteristics of patients with or without bleeding complications

	EDX group N = 153 Complication		*P* value	WFR group N = 103 Complication		*P* value
	Yes	No		Yes	No	
No. of patient	12	141		9	94	
Age, years, median (IQR)	62 (51‐75)	66 (55‐72)	.724	72 (67‐73)	67 (59‐73)	.228
≧70	5 (42%)	50 (36%)		5 (56%)	38 (40%)	
＜70	7 (58%)	91 (64%)		4 (44%)	56 (60%)	
Female sex	4 (33%)	44 (31%)	.554	4 (44%)	22 (23%)	.161
Body weight, kg, (IQR)	60 (55‐67)	65 (57‐74)	.309	61 ± 13	67 ± 12	.161
≧60	6 (50%)	93 (66%)		5 (56%)	67 (71%)	
＜60	6 (50%)	48 (34%)		4 (44%)	27 (29%)	
Height, cm	164 ± 8	165 ± 9	.774	159 ± 10	165 ± 9	.088
Paroxysmal AF	11 (92%)	123 (87%)	.545	6 (67%)	59 (63%)	.562
Nonparoxysmal AF	1 (8%)	18 (13%)		3 (33%)	35 (37%)	
Persistent AF	0 (0%)	16 (11%)		3 (33%)	18 (19%)	
Long persistent AF	1 (8%)	2 (2%)		0(0%)	17 (18%)	
Past medical history						
Hypertension	3 (25%)	66 (47%)	.145	4 (44%)	44 (47%)	.587
Diabetes mellitus	1 (8%)	19 (14%)	.516	4 (44%)	21 (22%)	.143
Congestive heart failure	0(0%)	5 (4%)	.661	1 (11%)	11 (12%)	.719
Previous stroke/TIA	0 (0%)	3 (2%)	.781	0 (0%)	7 (7%)	.517
CKD (eGFR <50)	0 (0%)	11 (8%)	0.603	4 (44%)	11 (12%)	.024
CHADS₂ score						
0‐1	10 (83%)	114 (81%)	.595	7 (78%)	64 (68%)	.428
≧2	2 (17%)	27 (19%)		2 (22%)	30 (32%)	
HAS‐BLED score						
0‐2	12 (100%)	139 (99%)	.849	6 (67%)	92 (98%)	.004
≧3	0 (0%)	2 (1%)		3 (33%)	2 (2%)	
Echographic parameters						
LA diameter, mm	37 ± 7	38 ± 6	.750	38 ± 5	41 ± 7	.092
LVEF, %, (IQR)	63 (56‐72)	63 (58‐66)	.623	63 (61‐65)	63 (53‐65)	.684
BNP, pg/mL, (IQR)	20 (9‐62)	39 (20‐74)	.196	116 (20‐410)	59 (29‐110)	.252
eGFR, mL/min/1.73 m^2^	73 ± 13	70 ± 14	.438	45 ± 28	67 ± 17	.001
PT‐INR	N.A	N.A	N.A	2.3 ± 0.4	2.2 ± 0.5	.518
Procedure values						
Mean ACT (sec)	322 ± 33	327 ± 26	.738	337 ± 33	336 ± 28	.849
Injected heparin dose, U, (IQR)	13 300 (10 500‐15 000)	13 000 (11 100‐15 000)	.666	7000 (5900‐8500)	8000 (7000‐10 000)	.04
Protamine sulfate use (%)	5 (42%)	42 (30%)	.290	3 (33%)	24 (26%)	.442

Abbreviations: ACT, activated clotting time; AF, atrial fibrillation; BNP, brain natriuretic peptide; EDX, edoxaban; eGFR, estimate glomerular rate; IQR, interquartile range; LA, left atrium; LVEF, left ventricular ejection fraction; PT‐INR, prothrombin time‐international normalized ratio; TIA, transient ischemic attack; WFR, warfarin.

## 4 DISCUSSION

The main findings of the present, real‐world, observational study were as follows. Firstly, uninterrupted anticoagulation treatment with EDX or WFR during the peri‐procedural period of CA prevented the occurrence of thromboembolism in real‐world patients receiving CA for AF. Secondly, uninterrupted anticoagulation therapy with EDX or WFR was overall safe based on the low incidence of serious bleeding complications noted in both groups. Thirdly, patients with renal failure (eGFR <30) and/or higher bleeding risk (HAS‐BLED score ≥3) who received uninterrupted anticoagulation therapy with WFR were at a markedly high risk of bleeding complications.

It has been shown that uninterrupted anticoagulation treatment with WFR was effective for the prevention of peri‐procedural complications in CA, compared with an interrupted anticoagulation strategy.^12^ Recently, DOACs emerged as an alternative to WFR, and the safety and efficacy of those drugs have also been demonstrated in clinical studies.^13^, ^14^, ^15^ The ELIMINATE AF trial was a randomized control trial comparing uninterrupted treatment with EDX vs WFR in patients undergoing CA for AF.^7^ The primary endpoint (death, stroke, or major bleeding defined by the International Society on Thrombosis and Haemostasis) did not significantly differ between the EDX and WFR groups (2.7% vs 1.7%, respectively; hazard ratio: 1.6; 95% confidence interval: 0.44‐5.78). In that study, only one thromboembolic complication occurred in one patient (0.3%) in the EDX group. There was no death reported in either of the groups. Clinically relevant nonmajor bleeding occurred in 7.9% and 3.6% of the patients in the EDX and WFR groups, respectively. However, the data were not statistically analyzed.

In the present observational study, we examined the real‐world Japanese population, including patients with mechanical heart valves, ischemic heart disease receiving dual antiplatelet therapy, and kidney failure undergoing hemodialysis. These patients were excluded from the randomized clinical studies, such as the ELIMINATE AF trial. Our real‐world observational study confirmed the efficacy of uninterrupted anticoagulation therapy with EDX or WFR during CA for AF in such high‐risk patients (thromboembolism: 0%, in both groups). The incidence of major bleeding complications in the EDX and WFR groups was only 0.7% and 2.9%, respectively, warranting the safety of uninterrupted anticoagulation treatment in real‐world practice. In our study, the total rate of major and minor bleeding complications in the EDX and WFR groups was 7.8% and 8.7%, respectively. The latter rate was relatively higher than that reported in the ELIMINATE AF trial, probably reflecting a higher bleeding risk in the WFR group in our study. The Fushimi registry, a Japanese all‐comer registry consisting of 3,282 patients with AF, showed a similar trend to that observed in our study in terms of patient characteristics: higher comorbidity rates (diabetes mellitus, congestive heart failure, chronic kidney disease, and nonparoxysmal AF) and higher HAS‐BLED and CHADS_2_ scores in the WFR group compared with the DOAC group, and the absence of an oral anticoagulant group.^16^ Thus, we believe that the results of our study can be applied to the real‐world clinical practice.

In our study, it was also intriguing that the total rate of major and minor bleeding complications was markedly high in patients in the WFR group with an eGFR <30 (75%) or a HAS‐BLED score ≥3 (60%), while those of others (eGFR = 30‐49, eGFR ≥50, or HAS‐BLED score ≤2) were all <10% regardless of the administered anticoagulation agent (Figure 1). This indicates the need for a different peri‐procedural anticoagulation strategy for the treatment of patients with severe kidney dysfunction. Previous studies also showed that patients with chronic kidney disease receiving therapy with WFR had an increased risk of bleeding.^17^, ^18^ In addition, Yasaka et al demonstrated that patients with PT‐INR <1.6 and >2.6 were associated with an increased rate of bleeding and thromboembolic events.^19^ Moreover, a meta‐analysis showed that low‐intensity therapy with WFR (PT‐INR: 1.5‐2.0) decreased the risk of bleeding events without statistically increasing the risk of thromboembolic complications compared with standard‐intensity therapy with WFR (PT‐INR: 2.0‐3.0).^20^ In our patients who experienced bleeding complications, the preprocedural values of PT‐INR were within a higher range: 2.6‐3.0 and 2.1‐2.6 in those aged <70 years and ≥70 years, respectively. Therefore, more strict control of preprocedural anticoagulation conditions (eg, PT‐INR of 2.1‐2.6 and 1.6‐2.1 for patients aged <70 years and ≥70 years, respectively) may reduce the risk of bleeding complications in Japanese patients with severely impaired renal function.

Apart from the type of anticoagulation therapy, the timing of treatment also affects the rate of complications during the CA period. In a single‐arm observational study of patients with AF, Takahashi et al showed the efficacy and safety of uninterrupted EDX administered once in the morning, with one dose delayed on the procedural day. The results showed a low composite incidence of thromboembolism and major bleeding (0.2%).^21^ Hirao et al reported that administration of rivaroxaban within 8 hours prior to the CA procedure was associated with a higher incidence of major bleeding than administration 8‐28 hours prior to the CA procedure (18% vs 0%, respectively, *P* = .026).^22^ In the ELIMINATE AF trial, all patients with uninterrupted EDX therapy received the medicine in the evening (after the CA procedure), and reported a relatively low incidence of major bleeding (2.7%).^7^ In the present study, EDX was administered within 8 hours prior to or after the procedure at the operators' discretion. As shown in Table 3, the timing of EDX administration (pre‐CA or post‐CA) did not significantly affect the incidence of bleeding complications. However, the only one event of major bleeding recorded in the EDX group occurred in a patient who received pre‐CA treatment. In addition, more heparin was needed to maintain an ACT of 300‐350 seconds in the EDX group than the WFR group. A previous study revealed that more heparin was needed in patients with anticoagulation therapy using EDX than those using WFR. This study indicated that the difference in the half‐lives of WFR and EDX affects the ACT.^23^ Moreover, similar results have been reported for apixaban, rivaroxaban, and dabigatran.^24^ Our study produced similar results and supported the findings of other studies.

### 4.1 Limitations

There were several limitations in this study. Firstly, this was a retrospective, two‐center experience, observational study involving a relatively small number of patients. However, our study could show clinically relevant results with statistical significance, as other studies enrolling a relatively small number of patients.^15^, ^25^ Secondly, there could be an operator bias. The operators' experience with CA ranged from at least 100 to >2000 procedures. The rates of procedural complications, such as cardiac tamponade and false femoral aneurysm, are associated with the skill of an operator. Thirdly, patients with insufficient adherence to treatment were excluded from the analysis, which may have induced a patient‐selection bias.

## 5 CONCLUSION

This real‐world observational study confirmed the safety and efficacy of uninterrupted anticoagulation treatment during the peri‐procedural period of CA for AF. Uninterrupted therapy with EDX or WFR resulted in lower rates of thromboembolic and bleeding complications. Patients with severely impaired renal function and/or a higher bleeding risk during uninterrupted therapy with WFR were at a prominent risk of bleeding complications. Therefore, utmost attention should be paid in the treatment of these patients to prevent bleeding complications.

## CONFLICT OF INTEREST

All authors have no conflict of interest relevant to the topic of this manuscript.

## Supporting information

Fig S1
