# Supplementary figures and images for: Safety and efficacy of uninterrupted treatment with edoxaban or warfarin during the peri‐procedural period of catheter ablation for atrial fibrillation
Source: J Arrhythm. 2020 Apr 26;36(4):634–41. doi: 10.1002/joa3.12351 (PMC7411202; doi:10.1002/joa3.12351)

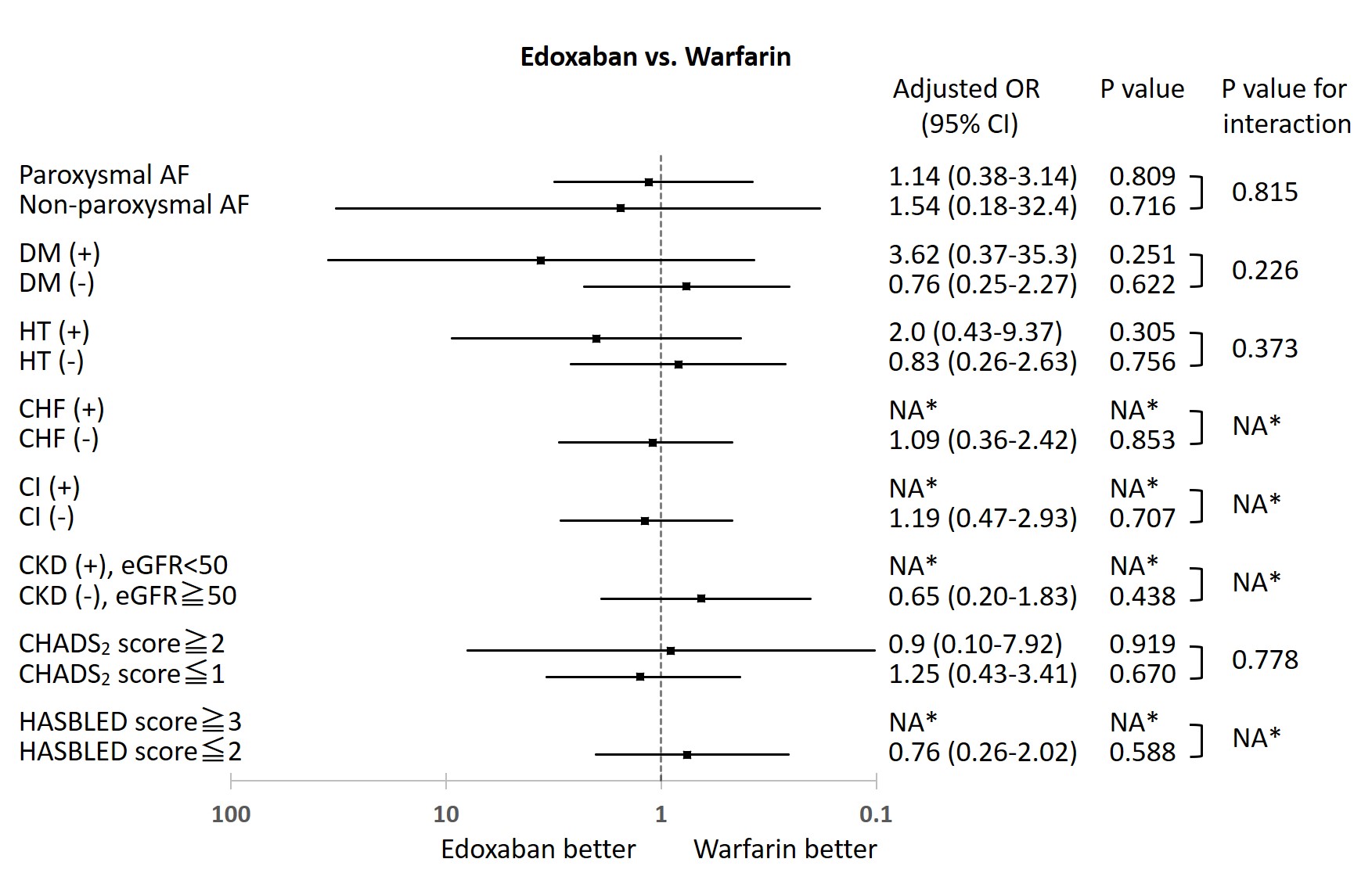

Supplement: Supplementary file 1 — Fig S1 [file JOA3-36-634-s001.jpg]
